# Identification of a Mutant PfCRT-Mediated Chloroquine Tolerance Phenotype in *Plasmodium falciparum*

**DOI:** 10.1371/journal.ppat.1000887

**Published:** 2010-05-13

**Authors:** Stephanie G. Valderramos, Juan-Carlos Valderramos, Lise Musset, Lisa A. Purcell, Odile Mercereau-Puijalon, Eric Legrand, David A. Fidock

**Affiliations:** 1 Department of Microbiology and Immunology, Columbia University Medical Center, New York, New York, United States of America; 2 Department of Microbiology and Immunology, Albert Einstein College of Medicine, Bronx, New York, United States of America; 3 Reference Centre for Plasmodium Chemoresistance in French Guiana and West Indies (CNRCP), Institut Pasteur de la Guyane, Cayenne, French Guiana; 4 Parasite Molecular Immunology, CNRS URA 2581, Institut Pasteur, Paris, France; 5 Department of Medicine, Columbia University Medical Center, New York, New York, United States of America; Case Western Reserve University, United States of America

## Abstract

Mutant forms of the *Plasmodium falciparum* transporter PfCRT constitute the key determinant of parasite resistance to chloroquine (CQ), the former first-line antimalarial, and are ubiquitous to infections that fail CQ treatment. However, treatment can often be successful in individuals harboring mutant *pfcrt* alleles, raising questions about the role of host immunity or pharmacokinetics vs. the parasite genetic background in contributing to treatment outcomes. To examine whether the parasite genetic background dictates the degree of mutant *pfcrt*-mediated CQ resistance, we replaced the wild type *pfcrt* allele in three CQ-sensitive strains with mutant *pfcrt* of the 7G8 allelic type prevalent in South America, the Oceanic region and India. Recombinant clones exhibited strain-dependent CQ responses that ranged from high-level resistance to an incremental shift that did not meet CQ resistance criteria. Nonetheless, even in the most susceptible clones, 7G8 mutant *pfcrt* enabled parasites to tolerate CQ pressure and recrudesce *in vitro* after treatment with high concentrations of CQ. 7G8 mutant *pfcrt* was found to significantly impact parasite responses to other antimalarials used in artemisinin-based combination therapies, in a strain-dependent manner. We also report clinical isolates from French Guiana that harbor mutant *pfcrt*, identical or related to the 7G8 haplotype, and manifest a CQ tolerance phenotype. One isolate, H209, harbored a novel PfCRT C350R mutation and demonstrated reduced quinine and artemisinin susceptibility. Our data: 1) suggest that high-level CQR is a complex biological process dependent on the presence of mutant *pfcrt*; 2) implicate a role for variant *pfcrt* alleles in modulating parasite susceptibility to other clinically important antimalarials; and 3) uncover the existence of a phenotype of CQ tolerance in some strains harboring mutant *pfcrt*.

## Author Summary

*Plasmodium falciparum* resistance to the antimalarial drug chloroquine has been found to result primarily from point mutations in PfCRT, which provide a highly sensitive marker of *in vivo* treatment failure and *in vitro* resistance. Debate has nonetheless continued about the singular role of mutant PfCRT and the contribution of the parasite genetic background. To address this, we have generated recombinant *P. falciparum* lines expressing a mutant *pfcrt* allele, or the reference wild type allele, in three distinct chloroquine-sensitive strains. Their analysis reveals a spectrum of responses ranging from high-level resistance to a previously unrecognized tolerance phenotype. The latter is characterized by virtually unchanged chloroquine IC_50_ values, significantly elevated IC_90_ values, and the ability to recrudesce after exposure to drug concentrations that are lethal to chloroquine-sensitive parasites. This tolerance phenotype was also observed in an isolate from French Guiana, confirming its presence in malaria-endemic regions. Mutant PfCRT significantly affected parasite responses to other antimalarials, including ones used in artemisinin-based combination therapies, in a strain-dependent manner. Our data suggest that successful CQ treatment of drug-resistant parasites is dependent on both host immunity and the strain-dependent extent to which mutant *pfcrt* imparts resistance.

## Introduction

The massive use of chloroquine (CQ) in the 20^th^ century heralded substantial gains in the global fight against malaria. These advances were later lost as CQ resistance (CQR) arose and spread throughout malaria-endemic areas [1], [2]. Today, CQ and the alternative first-line antimalarial sulfadoxine-pyrimethamine have officially been mostly replaced by artemisinin-based combination therapies (ACTs) [3]. Nevertheless, CQ continues to be widely used in parts of sub-Saharan Africa at the household level, presumably because of its ability to provide temporary relief from symptoms for patients unable to afford ACTs or other expensive drugs [4], [5]. Recent findings also suggest the possibility of reintroducing CQ-based combination therapies into African regions where an extended hiatus from CQ use has led to the dominance of CQ-sensitive *Plasmodium falciparum* parasites that have outcompeted the less-fit CQ-resistant strains [6]. At the cellular level, CQ is thought to act by accumulating to low millimolar concentrations in the acidic digestive vacuole of asexual intra-erythrocytic *Plasmodium* parasites, wherein it interferes with the detoxification of iron-bound heme moieties produced as a result of hemoglobin degradation [7].

Clinical and epidemiological studies reveal that CQR emerged on very few occasions despite its abundant use, leading researchers to initially posit a multigenic basis of resistance [8]. This theory was challenged by the finding that CQR was inherited as a single locus in a genetic cross between the CQ-resistant Dd2 (Indochina) and the CQ-sensitive HB3 (Honduras) clones [9], [10]. The causal determinant in this locus was ultimately identified as the *P. falciparum* chloroquine resistance transporter (*pfcrt*), whose 49 kDa protein product PfCRT resides on the DV membrane [11], [12]. Comparison of the Dd2 and HB3 sequence revealed eight point mutations that all mapped to sites within or near several of the 10 putative transmembrane domains [11].

Quantitative trait loci analysis of the HB3×Dd2 cross has revealed that mutant *pfcrt* from the Dd2 parent accounts for >95% of the CQ response variation among the progeny [13]. Further evidence supporting *pfcrt* as the primary determinant of CQR has come from studies of culture-adapted field isolates, which show extensive linkage disequilibrium surrounding the *pfcrt* locus in CQ-resistant isolates [14]. These data suggest that strong selective sweeps drove mutant *pfcrt* through *P. falciparum* populations across the globe, a notion also supported by more recent studies of nucleotide diversity in geographically distinct strains [15], [16]. The PfCRT K76T mutation, ubiquitous to CQ-resistant strains, has proven to be a highly sensitive marker of CQR *in vitro* and is associated with a significantly increased risk of CQ treatment failure *in vivo* [17]–[19].

While these studies have demonstrated the primary importance of *pfcrt* in CQR, other evidence suggests that additional genes might contribute to the CQR phenotype. Most notably, a strain-dependent association has been demonstrated between mutant *pfcrt* and point mutations in *pfmdr1*. This may reflect parasite physiologic adaptations to counteract the fitness cost of mutant PfCRT, or an independent role for *pfmdr1* in CQR [1], [8], [19]–[22]. Nevertheless, even with identical *pfcrt* and *pfmdr1* alleles, large variations in response to CQ can exist, suggestive of a secondary effect of additional parasite factors [13], [23]–[25].

Clinically, resistance to CQ is graded by the World Health Organization ETF-LTF-ACPR system (corresponding to early treatment failure, late treatment failure, or adequate clinical and parasitological response), based on the time to manifest clinical or parasitological evidence of treatment failure [26]. Studies aimed at dissecting the roles of *pfcrt* and *pfmdr1* mutations in modulating the different grades of *in vivo* resistance have shown an increased risk of early treatment failure with PfCRT K76T, which in some reports is augmented with PfMDR1 N86Y [27], [28]. However, the PfCRT K76T molecular marker cannot reliably predict CQ treatment failure, revealing moderate specificity of this marker. Discordance between *in vitro* parasite responses and *in vivo* patient outcomes following CQ treatment can be as high as 20% [17], [29]. This discordance can be partially attributed to host and environmental factors, including patient immunity, individual pharmacokinetic differences, polyclonal infections, and limitations in obtaining repeated measurements of drug susceptibilities with patient isolates [30]. An additional explanation could be the variable presence of additional parasite determinants.

We have previously adopted allelic exchange strategies to show that different mutant *pfcrt* alleles could confer verapamil (VP)-reversible CQR in a single, defined genetic background, the CQ-sensitive strain GC03 [31]. A separate transfection-based study found that *pfcrt*-mediated CQR in two geographically distinct strains, Dd2 (from Indochina) and 7G8 (from Brazil), was entirely dependent on the presence of the K76T mutation [32]. These strains were chosen as they encode a PfCRT haplotype frequently observed in Africa and Asia (Dd2) or in Papua New Guinea, South America and India (7G8). Both alleles have been documented in multiple clinical trials to be highly specific for CQ treatment failures, with repeated evidence of significant selection for mutant *pfcrt* of either allelic type in early or late treatment failures. Frequencies of mutant alleles in those cases often attained 100% [17], [33]–[37]. Trials were conducted in Africa, Southeast Asia, South America or the Oceanic region.

Here, we have assessed the effect of mutant *pfcrt* on the CQ response of three CQ-sensitive strains. We also describe two isolates from French Guiana that provide clinical validation of our genetic investigations. Our data reveal the existence of a mutant PfCRT-mediated CQ tolerance phenotype in some strains of *P. falciparum*.

## Results

### Generation of recombinant lines expressing mutant *pfcrt*

**Figure 1:**
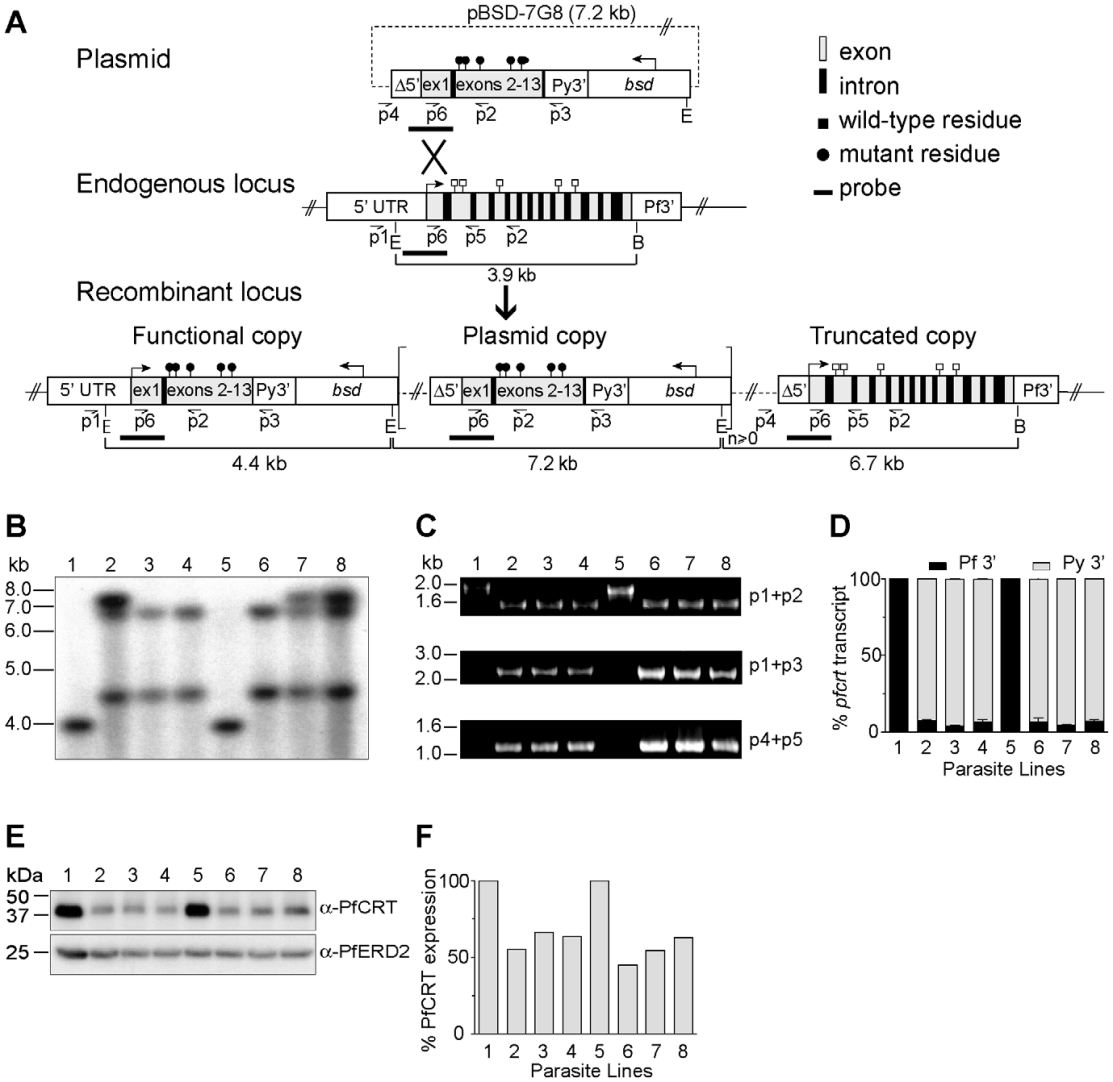
*pfcrt* allelic exchange strategy and molecular characterization of clones. (A) Schematic representation of single-site crossover between the transfection plasmid pBSD-7G8 and the endogenous *pfcrt* allele, leading to expression of a recombinant allele containing the 7G8 polymorphisms (black circles), transcribed from the endogenous 3.0 kb full-length promoter. In some parasites the downstream plasmid sequence integrated as tandem linear copies (delineated as square brackets with a copy number n≥0). The distal truncated locus harbored the Δ5′ UTR, which was previously found in luciferase assays to have minimal activity. E, *Eco*RI; B, *Bgl*II. (B) Southern blot hybridization of *Eco*RI/*Bgl*II–digested genomic DNA samples hybridized with a *pfcrt* probe from the 5′ UTR and exon 1 region (depicted in panel A). (C) PCR analyses of the recombinant clones and parental lines. (D) Transcript levels from the functional and truncated *pfcrt* loci (terminated by Py3′ and Pf3′ UTRs respectively in the case of the recombinant clones). Data are presented as a percentage of total *pfcrt* transcript levels normalized against the respective WT control (3D7 or D10). (E) Western blot analysis of recombinant and parental lines, probed with antibodies to PfCRT or the ER-Golgi marker PfERD2 [80]. (F) Signals were quantified, normalized against PfERD2, and expressed as a proportion of the signals obtained in the appropriate parental line. (B–F) Lanes: 1-3D7, 2-3D7^C^, 3-3D7^7G8-1^, 4-3D7^7G8-2^, 5-D10, 6-D10^C^, 7-D10^7G8-1^, and 8-D10^7G8-2^. GC03 clones were also confirmed by PCR, sequencing, and Southern hybridization, and were found to have similar levels of *pfcrt* RNA and protein expression as compared with the 3D7 and D10 clones (data not shown).

To define the impact of mutant *pfcrt* on CQ response in diverse genetic backgrounds, we developed an allelic exchange strategy based on a single round of homologous recombination and single-site crossover integration (Figure 1A), and applied this to the CQ-sensitive *P. falciparum* strains 3D7 (isolated in the Netherlands), D10 (Papua New Guinea), and GC03 (a progeny of the HB3×Dd2 genetic cross). This strategy differed from an earlier approach that required two rounds of allelic exchange to generate the desired recombinants [31]. Briefly, we constructed selectable transfection plasmids that contained a 2.9 kb *pfcrt* insert consisting of 0.5 kb of the endogenous 5′ untranslated region (UTR), exon 1, intron 1, and the remaining exons 2–13 (Figure 1A). This truncated 5′ UTR fragment (termed Δ5′) was previously observed by luciferase assays to give insignificant levels of activity (A. Sidhu, unpublished data). Single-site crossover between the *pfcrt* insert and the homologous *pfcrt* sequence upstream of codons 72–76 was predicted to replace the endogenous *pfcrt* gene with a recombinant allele harboring all the single nucleotide polymorphisms from the 7G8 or Dd2 *pfcrt* allele. Expression of this recombinant allele was driven by the endogenous full-length (3.0 kb) 5′ UTR and a previously characterized, functional 0.7 kb 3′ UTR (termed Py3′) from the *pfcrt* ortholog in *Plasmodium yoelii* [31]. In addition to these pBSD-7G8 and pBSD-Dd2 constructs, we also generated the control pBSD-GC03 plasmid that encoded the wild type (WT) *pfcrt* sequence in order to obtain recombinant control parasites.

3D7, D10 and GC03 parasites were transfected with the pBSD-7G8, pBSD-Dd2, or pBSD-GC03 plasmids and screened monthly by PCR for homologous recombination at the *pfcrt* locus. With the 7G8 and GC03 alleles, integration into the *pfcrt* locus was first detected within 60 days of electroporation, and subsequently cloned by limiting dilution. In contrast, the Dd2 allele failed to show PCR evidence of homologous recombination even after 200 days of continuous culture in 3 separate transfection experiments, suggesting that this allele was detrimental to the growth of 3D7 and D10 parasites (data not shown). Repeated efforts failed to transfect 7G8 and Dd2 *pfcrt* alleles into the CQ-sensitive strains MAD1 and Santa Lucia (from Madagascar and Santa Lucia, a kind gift of Drs Milijaona Randrianarivelojosia and Dennis Kyle respectively), as well as HB3. Recombinant parasites either never appeared following plasmid electroporation and drug selection, or the plasmids never integrated into the *pfcrt* locus.

Successful transfection of the 3D7, D10 and GC03 strains produced the recombinant mutant clones 3D7^7G8-1^, 3D7^7G8-2^, D10^7G8-1^, D10^7G8-2^, GC03^7G8-1^ and GC03^7G8-2^ (all generated from the plasmid containing the 7G8 *pfcrt* sequence) or the recombinant control clones 3D7^c^, D10^c^ and GC03^c^ clones (generated with the control plasmid harboring the WT *pfcrt* sequence; Table 1). Southern hybridization of *Eco*RI*/Bgl*II-digested genomic DNA samples with a *pfcrt* probe confirmed the expected recombinant locus, as evidenced by the loss of a 3.9 kb band present in the WT lines and the acquisition of 4.4 kb and 6.7 kb bands consistent with recombination in *pfcrt* (results shown for the 3D7 and D10 clones in Figure 1B). The 7.2 kb bands present in 3D7^C^, D10^7G8-1^, and D10^7G8-2^ were indicative of integration of tandem plasmid copies into the *pfcrt* locus.

**Table 1:** Summary of *pfcrt*-modified lines and reference strains.

			PfCRT haplotype (72–371)										PfMDR1 haplotype (86–1246)						Microsatellite marker			
Line	Parent	Transfection Plasmid	72	74	75	76	220	271	326	350	356	371	86	184	1034	1042	1246	*pfmdr1* copy number	TA81	TA87	ARA2	PfPK2
3D7			C	M	N	K	A	Q	N	C	I	R	N	Y	S	N	D	1	173	82	102	170
3D7^c^	3D7	pBSD-GC03	C	M	N	K	A	Q	N	C	I	R	N	Y	S	N	D	1	173	82	102	170
3D7^7G8-1,2^	3D7	pBSD-7G8	**S**	M	N	**T**	**S**	Q	**D**	C	**L**	R	N	Y	S	N	D	1	173	82	102	170
D10			C	M	N	K	A	Q	N	C	I	R	N	Y	S	N	D	1	170	82	102	164
D10^c^	D10	pBSD-GC03	C	M	N	K	A	Q	N	C	I	R	N	Y	S	N	D	1	170	82	102	164
D10^7G8-1,2^	D10	pBSD-7G8	**S**	M	N	**T**	**S**	Q	**D**	C	**L**	R	N	Y	S	N	D	1	170	82	102	164
GC03			C	M	N	K	A	Q	N	C	I	R	N	Y	S	**D**	D	1	182	95	102	161
GC03^c^	GC03	pBSD-GC03	C	M	N	K	A	Q	N	C	I	R	N	Y	S	**D**	D	1	182	95	102	161
GC03^7G8-1,2^	GC03	pBSD-7G8	**S**	M	N	**T**	**S**	Q	**D**	C	**L**	R	N	Y	S	**D**	D	1	182	95	102	161
7G8			**S**	M	N	**T**	**S**	Q	**D**	C	**L**	R	N	**F**	**C**	**D**	**Y**	1	170	98	98	173
G224			**S**	M	N	**T**	**S**	Q	**D**	C	**L**	R	N	**F**	S	**D**	**Y**	1	170	98	98	173
H209			**S**	M	N	**T**	**S**	Q	**D**	**R**	**L**	R	N	**F**	S	**D**	**Y**	1	176	98	98	173
HB3			C	M	N	K	A	Q	N	C	I	R	N	Y	S	**D**	D	1	182	92	102	193
Dd2			C	**I**	**E**	**T**	**S**	**E**	**S**	C	**T**	**I**	**Y**	Y	S	N	D	4	176	95	108	161

Transfection plasmids encoding for the chloroquine (CQ)-sensitive GC03 allele or the CQ-resistant 7G8 allele were transfected into CQ-sensitive parental strains 3D7, D10 and GC03 to generate the recombinant control and mutant lines. PfCRT and PfMDR1 haplotypes are shown for the polymorphic amino acid residues. Residues that differ from the wild-type sequence are shown in bold. *pfmdr1* copy number was determined by quantitative PCR. Results of genotyping with microsatellite markers are shown for each line.

We confirmed these recombination events using PCR analyses with a 5′ UTR-specific primer (p1) and an exon 5-specific primer (p2), which revealed a change in size from the 1.8 kb WT-specific band to a shorter 1.5 kb band in the recombinant controls and mutants reflecting the loss of introns 2–4 (Figure 1C). The recombinant controls and mutants also showed the acquisition of PCR bands specific for the full-length functional copy of the *pfcrt* locus (2.2 kb, p1+p3) and the downstream truncated copy (1.1 kb, p4+p5) (Figure 1C). Sequencing of these PCR products (data not shown) confirmed that the integration event placed the K76T mutation in the functional locus, and that the WT allele was displaced to the downstream non-functional locus. Reverse-transcriptase (RT)-PCR assays on synchronized ring stage RNA with primers specific to exons 2 and 5 (p6+p2) produced a single band corresponding to cDNA, with no evidence of genomic DNA contamination (data not shown). Sequence analysis of those products detected transcripts only from the functional recombinant locus (under the control of the 3.0 kb full-length 5′ UTR) and not from the downstream truncated locus (data not shown). No endogenous WT locus was detected in any recombinant clone.

To precisely assess the transcriptional status of the functional vs. the truncated *pfcrt* copies, we performed quantitative real-time RT-PCR utilizing primers specific for transcripts containing the Py3′ vs. Pf3′ UTRs respectively. Quantification of *pfcrt* steady state transcript levels was made by extrapolation from a standard curve generated from genomic DNA of D10^C^, which has a single copy of the pBSD-GC03 plasmid integrated into the *pfcrt* locus (Figure 1B). Results showed that transcription from the functional *pfcrt* allele with Py3′ accounted for 93–95% of the total *pfcrt* transcript within each line (Figure 1D). Western blot analysis showed that PfCRT protein levels in the recombinant 3D7 and D10 lines were 55–66% and 45–63% those observed in the parental controls respectively (Figures 1E and 1F). This finding of reduced *pfcrt* transcript and protein expression levels following allelic exchange is consistent with earlier *pfcrt* transfection studies [31], [32], [38]. Importantly, those studies have shown that reduced *pfcrt* expression in recombinant lines causes a concomitant reduction in CQ IC_50_ values, which thus become lower than the IC_50_ values observed in parasites harboring non-recombinant *pfcrt*. In our drug assays, the IC_50_ value refers to the drug concentration that inhibits incorporation of [^3^H]-hypoxanthine, a marker of *in vitro* parasite growth, by 50%.

### Mutant *pfcrt* is insufficient to confer high-level chloroquine resistance in the 3D7 and D10 genetic backgrounds

**Figure 2:**
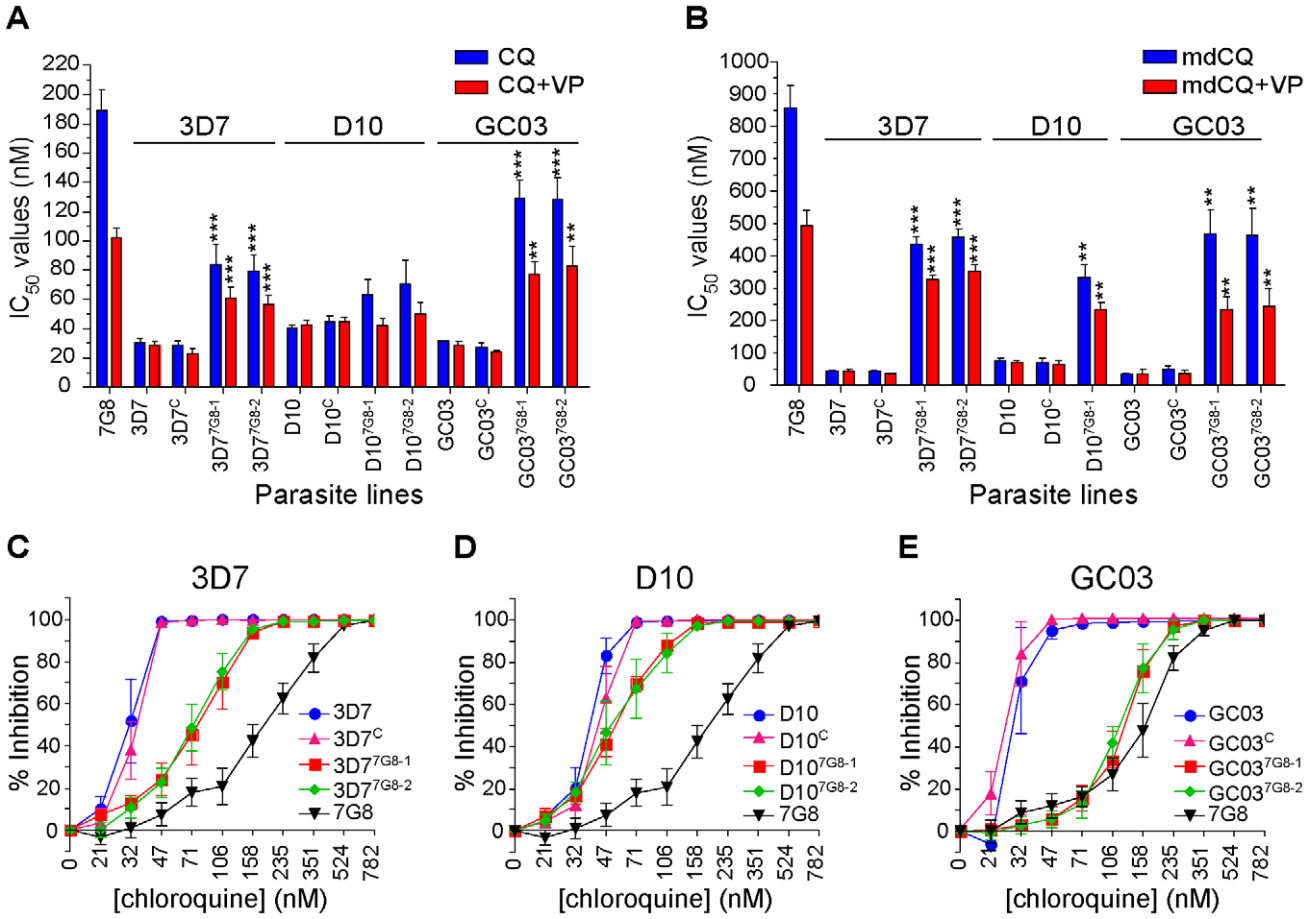
*In vitro* response of *pfcrt-*modified clones to chloroquine and its primary metabolite monodesethylchloroquine. *In vitro* [^3^H]-hypoxanthine incorporation assays were performed with the WT, control, and mutant *pfcrt* clones. All lines were tested in duplicate against CQ±VP and mdCQ±VP an average of 7 times (range 4–11; see summary in Table S1). Mean±SEM IC_50_ values are presented for (A) CQ and (B) its metabolite mdCQ. Statistical comparisons comparing mutant *pfcrt*-modified lines against recombinant control lines of the same genetic backgrounds were performed using one-way ANOVA with a Bonferroni post-hoc test. **P<0.01; ***P<0.001. (C–E) Percent inhibition of growth (shown as means±SEMs derived from all assays) across a range of CQ concentrations for (C) 3D7, (D) D10, and (E) GC03 lines.

Once the desired integration events were confirmed, we assessed the effect of mutant *pfcrt* on the CQ response in the recombinant lines. In the 3D7 background, mutant *pfcrt* was found to confer a 2.7-fold increase in CQ IC_50_ values (mean±SEM CQ IC_50_ values of 84±14 nM and 79±11 nM for 3D7^7G8-1^ and 3D7^7G8-2^ respectively) compared to the 3D7 recombinant control (29±2 nM, *P*<0.001; Figure 2A, Table S1). These values were 2.4-fold lower than the IC_50_ values for WT 7G8 (190±14 nM). For the D10 mutants, there was no significant increase in CQ IC_50_ values for D10^7G8-1^ and D10^7G8-2^ compared to D10^C^ (63±11 nM, 71±16 nM, and 45±3 nM, respectively, *P*>0.05).

When tested against the primary *in vivo* metabolite monodesethyl-chloroquine (mdCQ), a significant decrease in susceptibility was found in both genetic backgrounds. The 3D7 mutant clones demonstrated a 10-fold increase in mdCQ IC_50_ values compared to 3D7^C^ (*P<*0.001, Figure 2B). In comparison, the IC_50_ values for the D10^7G8-1^ mutant were 5-fold higher than D10^C^ (*P<*0.01, Figure 2B, D10^7G8-2^ was not tested). Nevertheless, the mdCQ IC_50_ values in both backgrounds were approximately 2–fold lower than those observed in WT 7G8, suggesting that mutant *pfcrt* was insufficient to confer high-level mdCQ resistance to 3D7 and D10 parasites.

These findings of a relatively moderate, strain-dependent decrease in CQ susceptibility in the 3D7 and D10 *pfcrt* mutants contrasted with our earlier observation that the introduction of 7G8 mutant *pfcrt* in the GC03 background resulted in CQ IC_50_ values >100 nM [31]. To directly compare the effects of mutant *pfcrt* between strains, and to assess for any potentially confounding differences in our transfection strategies, we generated recombinant control (GC03^C^) and mutant clones expressing the 7G8 allele (GC03^7G8-1^ and GC03^7G8-2^) using our single-round transfection strategy. These clones were confirmed by PCR, sequencing, and Southern hybridization, and were found to have similar levels of *pfcrt* RNA and protein expression compared to the 3D7 and D10 clones (data not shown). In the GC03 background, introduction of the 7G8 mutant *pfcrt* allele increased the CQ IC_50_ values 4.7-fold (*P*<0.001), from 27±3 nM for GC03^C^ to ∼130±8 nM for both recombinant clones (Figure 2A, Table S1), and increased the mdCQ IC_50_ values by 9-fold (*P*<0.01; Figure 2B). These determinations included four independent assays that directly compared GC03^7G8-1^ and GC03^7G8-2^ with the C6^7G8^ line. The latter was produced using our earlier *pfcrt* modification strategy involving consecutive rounds of allelic exchange [31]. C6^7G8^ also expresses the 7G8 *pfcrt* allele in the GC03 background, yet differs from the clones produced in the current study in that C6^7G8^ contains both the human dihydrofolate reductase and the *bsd* selectable markers, and lacks the 0.5 kb 5′UTR present in the downstream *pfcrt* loci in the GC03^7G8-1^ and GC03^7G8-2^ clones (see Figure 1A). Drug assays with these lines produced CQ IC_50_ values of 131±7, 129±8 and 130±7 nM for GC03^7G8-1^, GC03^7G8^ and C6^7G8^ respectively (Table S1). These results are comparable to our published data with C6^7G8^ (127±17 nM; [31]) and are consistent with both allelic exchange strategies producing the same CQ responses. Our data from all three strains also provide clear evidence that the degree of CQR conferred by mutant *pfcrt* is strain-dependent.

We also found that the genetic background influenced the degree of VP chemosensitization, a hallmark of *P. falciparum* CQR [39]. In 3D7 and D10, expression of mutant *pfcrt* conferred a VP reversibility of 24±1% and 28±1% (calculated as the mean±SEM of percent reversibility for all CQ and mdCQ values), compared to 44±2% for GC03 (Figure S1). Notably, significant VP reversibility occurred in the D10 mutants despite the lack of a significant increase in CQ IC_50_ values (Figure 2D, Table S1). By comparison, VP reversibility for 7G8 CQ and mdCQ responses was 46±3% (Figure 2B). This is lower than the degree of VP reversibility that results from expression of the Dd2 *pfcrt* allele [31], [40].

Analysis of the dose response curves generated during these studies revealed a more complex picture than was evident from the IC_50_ values alone. For all three genetic backgrounds, introduction of the 7G8 mutant allele into the CQ-sensitive strains caused a pronounced change in the slope of the dose-response profiles, with evidence of continued growth at high CQ concentrations (Figures 2C–E). This was particularly pronounced for the recombinant D10^7G8-1^ and D10^7G8-2^ lines, whose CQ IC_50_ values were similar to those of D10 and D10^C^, yet whose IC_90_ values (i.e. the drug concentrations that inhibited [^3^H]-hypoxanthine uptake into cultured parasites by 90%) were greatly elevated. Indeed, analysis of the CQ IC_90_/IC_50_ ratios for the lines in each genetic background revealed significant increases in the mean ratios of the mutant lines (Figure S2). For the 3D7 and D10 backgrounds in particular, the relatively modest increase in CQ IC_50_ values appeared to be compensated by an increased ability of these parasites to withstand high CQ concentrations.

### The genetic background dictates whether mutant *pfcrt* confers chloroquine resistance or tolerance

We posited that these elevated IC_90_ values imparted by mutant *pfcrt* subtly reflected a CQ tolerance phenotype. To test this, we assayed our lines for the ability to survive treatment with 50 nM CQ, a concentration that was lethal after three generations of exposure for all three WT strains, and 80 nM CQ, which substantially exceeded each of their CQ IC_90_ values (Table S1).

**Figure 3:**
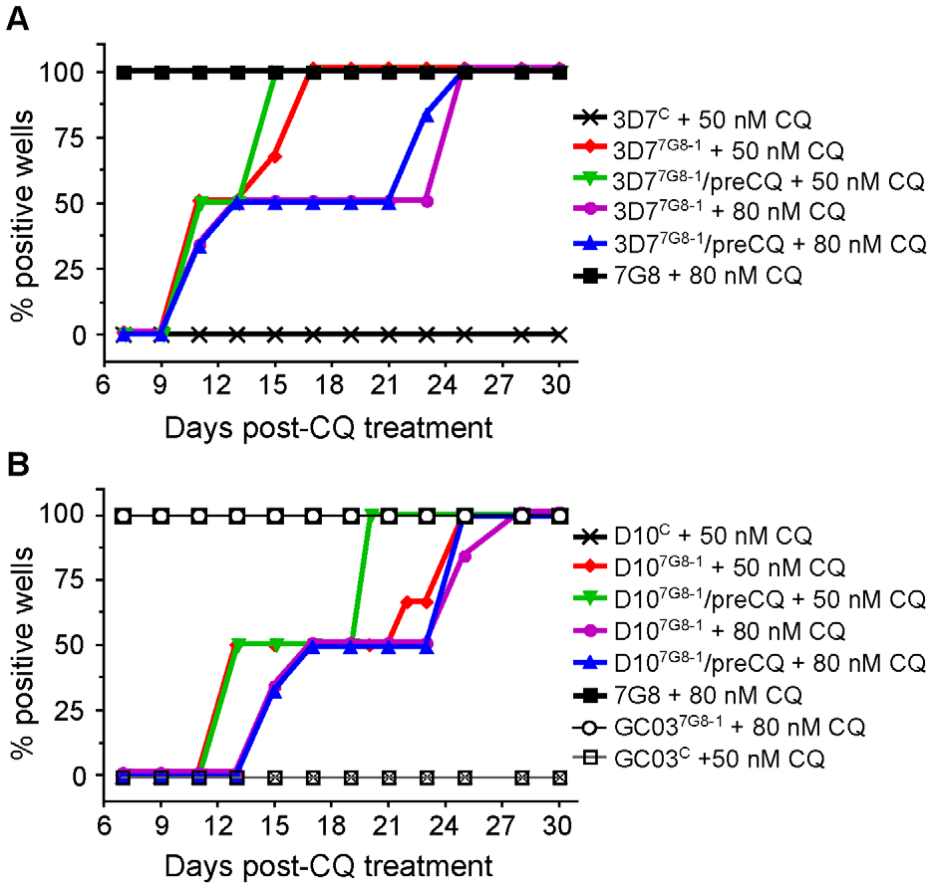
CQ recrudescence data for *pfcrt* -modified and parental clones. Lines were subjected to 50 nM or 80 nM CQ for 6 days and assayed for recrudescence every 2–3 days from days 7–30. Pooled data from two independent experiments were plotted as the percent of positive wells as a function of time post-CQ exposure. The panels show (A) 3D7 and (B) D10 clones and controls, including recombinant clones pretreated with 50 or 80 nM CQ. All no-treatment controls were positive on day 7 (as shown for 7G8+80 nM CQ), as was GC03^7G8-1^ treated with both 50 nM and 80 nM CQ.

Parental, control, and mutant lines were assayed for *in vitro* recrudescence (defined as 50% of cultures testing positive for growth) after a six-day exposure to CQ. The parental and recombinant control lines from the 3D7, D10, and GC03 backgrounds showed no signs of growth at 30 days post-exposure to 50 nM CQ (Figures 3A and 3B). In contrast, 3D7^7G8-1^ recrudesced at 9 and 13 days post-treatment with 50 nM and 80 nM CQ respectively (Figure 2A). We also tested 3D7^7G8-1^ that had been pretreated with 50 nM CQ for 3 generations approximately 30 days earlier (3D7^7G8-1^/preCQ), and observed similar rates of recrudescence. All untreated lines were positive at day 7, as was WT 7G8 that showed no inhibition of growth with 80 nM CQ treatment.

Although the introduction of mutant *pfcrt* resulted in no significant increase in CQ IC_50_ values in the D10 background, both D10^7G8-1^ and pretreated D10^7G8-1^/preCQ recrudesced at days 13 and 17 with treatment with 50 nM and 80 nM CQ, respectively (Figure 3B). In the GC03 background, GC03^7G8-1^ showed no inhibition of growth at 7 days with both 50 nM and 80 nM CQ treatments, reflecting the high-level CQR phenotype imparted by mutant *pfcrt* in this strain.

### Characterization of chloroquine-sensitive *P. falciparum* clinical isolates from French Guiana that possess mutant *pfcrt*

Given the evidence that mutant *pfcrt* was insufficient to confer CQR in all genetic backgrounds, we asked whether there were CQ-sensitive parasites harboring mutant *pfcrt* in the field. After an extensive search, this led to the identification of two clinical isolates from French Guiana that express the PfCRT K76T marker for CQR but are sensitive to CQ. These isolates, G224 and H209, were harvested in 2003 and 2004, respectively, and were genotyped at the *pfcrt* and *pfmdr1* loci. The PfCRT haplotype of G224 was found to be identical to that of 7G8, whereas H209 possessed a C350R mutation that has not been previously described (Table 1). Both G224 and H209 possessed a single copy of *pfmdr1* with the same haplotype that differed from 7G8 only at position 1034. Western blot analyses revealed equivalent levels of PfCRT expression compared to 7G8 (data not shown).

**Figure 4:**
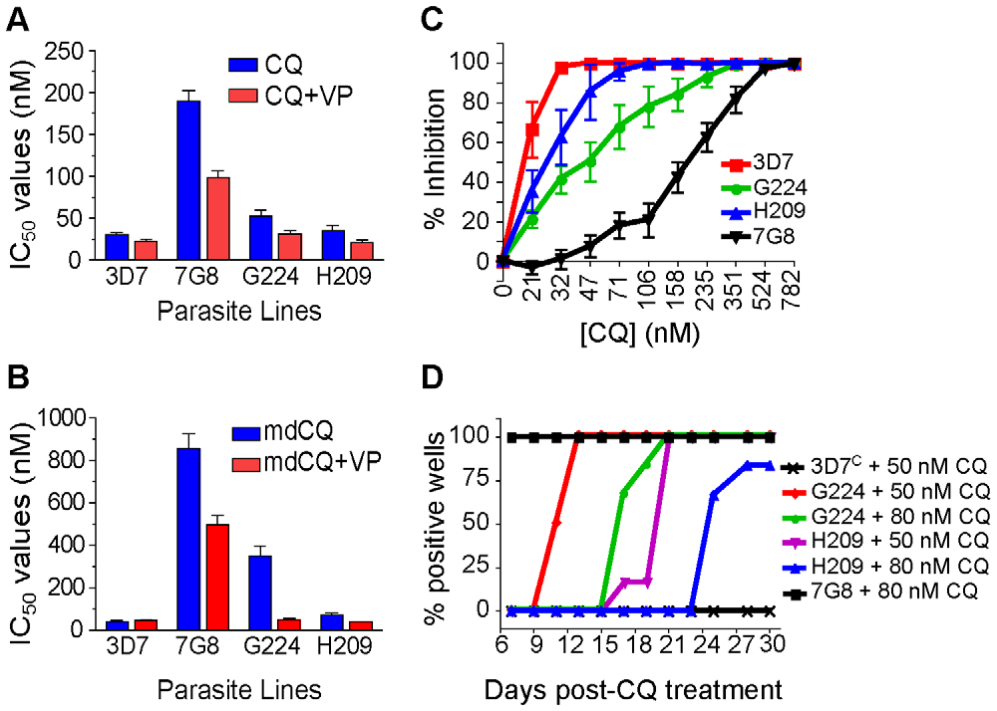
Characterization of the French Guiana isolates G224 and H209. (A–B) *In vitro* [^3^H]-hypoxanthine incorporation assays against CQ±VP (A) and mdCQ±VP (B) were performed with a CQ-sensitive (3D7) and a CQ-resistant (Dd2) control line. All lines were tested in duplicate on average 6 times (range 3–8). Mean±SEM IC_50_ values were derived by linear extrapolation. (C) Percent inhibition of growth (means±SEM derived from all assays) determined across a range of CQ concentrations. (D) Lines were exposed to 50 nM or 80 nM CQ for 6 days and assayed for recrudescence every 2–3 days from days 7–30. All no-treatment controls were positive on day 7 (as shown for 7G8+80 nM CQ).

Drug susceptibility assays using CQ and mdCQ showed that these strains had low IC_50_ values for CQ (mean IC_50_ values of 52±8 nM and 35±7 nM for G224 and H209, respectively) and mdCQ (mean IC_50_ values of 349±46 nM and 70±9 nM) (Figures 4A and 4B, Table S1). Further, both G224 and H209 demonstrated VP reversibility of their CQ and mdCQ response (averaging 37% and 35%, respectively; Table S1, Figure S1). Analysis of the CQ inhibition curves revealed that the IC_90_ values were skewed towards the IC_90_ of 7G8 (Figure 4C), reminiscent of the effect seen in our 3D7 and D10 mutant *pfcrt* lines (Figures 2C and 2D). This was particularly pronounced for G224, whose IC_90_ for CQ was 123±27 nM. When tested for *in vitro* recrudescence after a 6-day exposure to CQ, G224 recrudesced at days 11 and 17 when treated with 50 nM and 80 nM CQ respectively (Figure 3D). Interestingly, H209 showed recrudescence at days 21 and 25 for 50 nM and 80 nM CQ respectively, despite having a very low CQ IC_90_ value of 44±7 nM.

### The genetic background also determines the effect of mutant *pfcrt* on response to other antimalarials

To test whether the host strain also influenced the effect of mutant *pfcrt* on parasite response to other drugs, particularly those currently used in ACTs, we tested our lines against quinine (QN), artemisinin (ART), monodesethyl-amodiaquine (mdADQ, the potent *in vivo* metabolite of amodiaquine), lumefantrine (LMF), and piperaquine (PIP). The responses of the French Guiana isolates G224 and H209 were also assessed.

**Figure 5:**
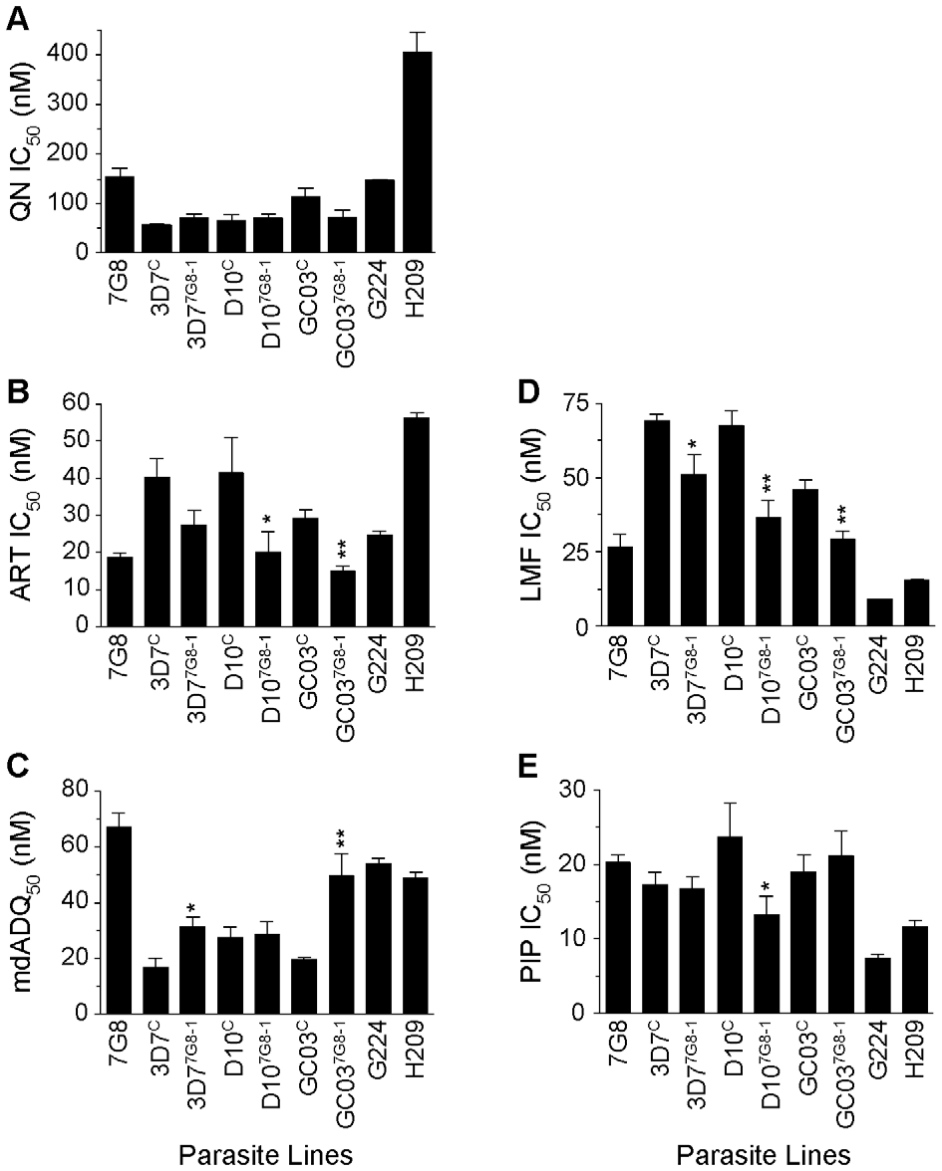
*In vitro* response of *pfcrt*-modified clones and French Guiana isolates to clinically important antimalarials. *In vitro* [^3^H]-hypoxanthine incorporation assays were performed in duplicate an average of 6 separate times (range 3–12 independent assays). Mean±SEM IC_50_ values are presented for (A) quinine (QN), (B) artemisinin (ART), (C) monodesethyl-amodiaquine (mdADQ), (D) lumefantrine (LMF), and (E) piperaquine (PIP). Statistical comparisons comparing mutant *pfcrt*-modified lines against recombinant control lines of the same genetic backgrounds were performed using unpaired student *t* tests. *P<0.05; **P<0.01. For brevity, a single recombinant mutant clone is presented for each host strain. Results from additional recombinant mutant lines are included in Table S1.

In the 3D7, D10 and GC03 backgrounds, we observed no effect of mutant *pfcrt* on QN response (Figure 5A, Table S1). Interestingly, the highest QN IC_50_ values were observed with H209, which showed a moderately high level of resistance (405±40 nM). When tested against ART, introduction of mutant *pfcrt* showed a significant 2–fold decrease in IC_50_ values in the D10 and GC03 backgrounds, when compared to recombinant clones expressing WT *pfcrt* (*P*<0.05 and *P*<0.01 respectively; Figure 5B). 3D7^7G8-1^ also yielded a 33% lower ART IC_50_ compared to the 3D7^C^ control, however this did not attain statistical significance (*P* = 0.06). Again, the highest ART IC_50_ values were observed with H209 (Table S1). For mdADQ, 3D7^7G8-1^ had a 1.5-fold increase in IC_50_ value compared to 3D7^C^ (*P*<0.05), and GC03^7G8-1^ showed an even more pronounced (2.6-fold) increase compared to GC03^C^ (*P*<0.01; Figure 5C). There was no effect of mutant *pfcrt* on mdADQ response in the D10 background. With this drug, G224 and H209 were both moderately resistant, as was 7G8.

Introduction of mutant *pfcrt* was also found to confer significantly increased sensitivity to LMF in all three strains, equating to a 23%, 44%, and 35% decrease in IC_50_ values for the 3D7, D10, and GC03 backgrounds respectively (Figure 5D). H209 was also found to be less susceptible to LMF than was G224, mirroring their responses to QN and ART. Finally, we found that mutant *pfcrt* had a significant effect on PIP response only in the D10 background, in which D10^7G8-1^ was 1.7-fold less sensitive than D10^C^ (*P*<0.05). G224 and H209 were found to be 2.7- and 1.7-fold more sensitive to PIP when compared to 7G8.

## Discussion

Here, we provide evidence that the genetic background of *P. falciparum* determines whether expression of mutant *pfcrt* allele confers a full CQR phenotype, as defined by CQ IC_50_ values that exceed the *in vitro* CQR threshold [30], or instead mediates increased tolerance to CQ, as evidenced by dose-response shifts manifesting primarily at the IC_90_ level. All our recombinant clones expressing mutant *pfcrt* recrudesced *in vitro* after being exposed for three generations to concentrations of CQ that were uniformly lethal to CQ-sensitive parasites; however the rate of recrudescence varied with the genetic background. The GC03 mutant lines, which had the highest CQ IC_50_ values, showed no growth inhibition. In contrast, the *pfcrt-*mutant lines generated in the 3D7 and D10 backgrounds, as well as the clinical isolates G224 and H209, required 1–3 weeks for the detection of recrudescent parasites.

Based on our findings, we propose that IC_50_ values, which typically constitute the sole measurement of CQ response *in vitro*, adequately identify high-level CQR but are insufficient to detect strains that have low-level resistance or manifest tolerance to CQ. Instead, our data suggest that accurate determinations of IC_90_ values provide a more predictive measure of whether parasites can recrudesce in the presence of CQ concentrations that are lethal to drug-sensitive parasites, a trait that we here refer to as CQ tolerance. Tolerance is also apparent in decreased parasite susceptibility to the primary drug metabolite mdCQ. We posit that *pfcrt-*mediated CQ tolerance might be an important component of late treatment failures in patients. These are classified as cases where symptoms occur during a follow-up period of 4–28 days post CQ treatment, or asymptomatic infection appearing 7–28 days post-treatment (see the WHO 2006 publication on malaria treatment: http://whqlibdoc.who.int/publications/2006/9241546948_eng_full.pdf). In contrast, early treatment failures might result more often from infections with parasites in which mutant *pfcrt* exerts a higher degree of CQR. Early treatment failures are classified as the development of clinical or parasitological symptoms during the first three days following CQ treatment. We note that clinically, care must be taken when evaluating early failures, as these can also include patients that respond relatively slowly to treatment yet progress to full cure. Moreover, the joint effects of low-level CQ resistance reported here and acquired protective immunity might help explain why CQ treatment can successfully cure some infections harboring mutant *pfcrt* parasites in semi-immune individuals [1], [18]. The importance of immunity in shaping the host's ability to resolve drug-resistant infections harboring mutant *pfcrt* was first demonstrated in work from Mali that found that successful CQ treatment of *pfcrt* mutant parasites was strongly dependent on age, a known surrogate for protective immunity in endemic areas [18]. These data complement other observations in the malaria literature indicating that the immune response can allow a relatively ineffective drug to clear an infection, and even at times clear infections without therapy [41], [42]. Our data extend these reports by suggesting that successful CQ treatment of drug-resistant parasites is dependent both on the level of host immunity and the strain-dependent extent to which mutant *pfcrt* imparts CQR.

A review of our CQ IC_50_ data reveals a relatively weak effect of mutant *pfcrt*, which attained the widely used *in vitro* CQR threshold of 80–100 nM only for GC03 (Figure 2, Table S1). This threshold, however, was based on studies with field isolates [30] and does not readily extrapolate to our *pfcrt-*modified parasite lines. Our data (Figure 1) show that these lines underexpress *pfcrt*, a consequence of allelic exchange into this locus that was earlier shown to cause artificially low CQ IC_50_ values whose level of reduction was concordant with the degree of reduced expression [31], [32], [38]. In our current study, the importance is not the absolute levels of CQR that we measured, but rather the finding that the genetic background of CQ-sensitive strains dictates a spectrum of mutant *pfcrt*-mediated changes in CQ response that ranges from tolerance to high-level resistance.

We note that our data were obtained with the 7G8 *pfcrt* allele, which is known to have appeared independently in South America and the Oceanic region in or near Papua New Guinea and has recently spread throughout India [43]. The 7G8 haplotype (C72S/K76T/A220S/N326D/I356L) shares only two mutations (K76T/A220S) with the Dd2 haplotype (I74E/N75E/K76T/A220S/Q271E/N326S/I356T/R371I) that is common to Africa and SE Asia [11], [14], [44]. Our earlier allelic exchange studies on recombinant lines generated in the GC03 strain found that the 7G8 *pfcrt* haplotype confers a lower degree of resistance than that imparted by the Dd2 allele (averaging 15% and 45% less or CQ and mdCQ respectively). This was consistent with the intrinsic differences observed between the parental 7G8 and Dd2 strains [31]. It is possible that in the D10 and 3D7 strains, higher degrees of resistance might have been observed with the Dd2 allele, however we were unable to test this. We note that D10 originates from Papua New Guinea where the 7G8 allele is highly prevalent, and our lack of success with introducing the Dd2 *pfcrt* allele into either this strain or 3D7 suggests a physiologic context that precludes expression and normal viability. Other evidence of a fitness cost imparted by the Dd2 allele comes from studies in Malawi showing that this allele is progressively lost from the parasite population in the absence of sustained CQ pressure [45], [46].

Field studies have sometimes reported discordance in the association of K76T and *in vitro* CQR, suggesting the contribution of other genetic loci [24], [47]–[49]. However, the interpretation of these results has been confounded by potential inaccuracies stemming from measuring one-time drug responses from frequently polyclonal fresh patient isolates. Our study provides, to the best of our knowledge, the first report of culture-adapted, monoclonal isolates that harbor mutant *pfcrt* and that, based on multiple drug susceptibility assays, show low CQ IC_50_ values that fail to meet the standard criteria for CQR. These findings, obtained with the G224 and H209 isolates from French Guiana, therefore provide indisputable evidence that mutant *pfcrt* is insufficient to confer CQR to all genetic backgrounds. Nevertheless, both isolates exhibited tolerance to high CQ concentrations and recrudesced under CQ pressure. Microsatellite typing revealed a close genetic similarity between G224 and 7G8 (Table 1), with the exception of the residue at PfMDR1 position 1034 that could potentially affect CQ response [22], [50].

Of particular interest, H209 was highly sensitive to CQ and yet demonstrated delayed recrudescence (Figure 4). This might in part be attributable to the PfCRT C350R charge substitution in transmembrane domain 9, a region postulated to function in substrate binding and translocation [51]. Studies are underway to introduce the H209 *pfcrt* allele, encoding the C350R mutation, into GC03 parasites to compare these to the GC03^7G8^ parasites whose expressed *pfcrt* allele differs only at codon 350 (Table 1). We note that an adjacent charge substitution at residue 352 (Q352K/R) was previously selected by QN pressure in a CQ-resistant line, with a concomitant reversion to CQ-sensitivity [52]. The H209 line also showed elevated IC_50_ values for QN, as well as ART, when compared to G224 and 7G8 (Figure 4). Of note, QN-doxycycline, and more recently artemether-LMF, have been implemented as first line antimalarials in French Guiana since the cessation of CQ use for the treatment of *P. falciparum* malaria in the mid 1990s [53]. Indeed, a recent report from French Guiana documented the existence of several field isolates with elevated artemether IC_50_ values (>30 nM in 7 of 289 isolates), suggesting decreased susceptibility to this agent [54]. G224 was tested at that time and found to have an artemether IC_50_ value of ∼1 nM. H209, which yielded artemisinin IC_50_ values two-fold higher than G224 (Table S1), was isolated one year later. Our subsequent studies reveal comparable IC_50_ values between these two lines with the more potent clinical derivatives artemether, artesunate and artemether (values provided in Table S1).

ACTs are rapidly assuming the role of first line antimalarials around the world [55]. Our studies with isogenic *pfcrt*-modified lines confirm previous reports that mutations in PfCRT can significantly affect parasite susceptibility to many of the antimalarials that constitute these ACTs [19], [56], and provide evidence that for certain drugs this effect is strain-dependent (Figure 5). In the case of the fast-acting ART, all three strains displayed enhanced susceptibility upon introduction of mutant *pfcrt*. With the amodiaquine metabolite mdADQ, elevated IC_50_ values were noted in two of the three recipient strains, supporting earlier epidemiological evidence that mutant PfCRT might contribute to a multigenic basis of amodiaquine resistance ([57]–[59]; see below). The opposite effect was observed with the bisquinoline PIP, which is highly effective against CQ-resistant strains of *P. falciparum* [60], and for which we observed a strain-dependent increase in susceptibility. For LMF, significantly enhanced susceptibility was observed in all three genetic backgrounds, supporting recent field studies [59], [61]. The generally enhanced potency of LMF and artemisinin derivatives against mutant *pfcrt* parasites bodes well for the widely used LMF-artemether co-formulation. The enhanced susceptibility conferred by the mutant *pfcrt* 7G8 allele to the ACT partner drugs LMF and PIP, but not amodiaquine, has potentially important implications in regional antimalarial drug policy.

Our *pfcrt* and CQ data speak to a requirement for additional parasite factors that, at least in some strains, either augment the level of PfCRT-mediated CQR or on the contrary, create an intracellular physiologic environment in which PfCRT is unable to exert its full capacity to dictate CQR [62], [63]. *pfmdr1* would appear to be one gene that contributes to this strain-dependent effect. Transfection-based studies have shown that in CQ-resistant strains that harbor mutant *pfcrt*, mutations in *pfmdr1* can contribute to elevated CQ IC_50_ values, but only in a subset of strains. Mutant *pfdmr1* alone shows no effect on CQ response in sensitive parasites harboring wild-type *pfcrt* [19], [20]. Evidence from CQ treatment trials in African, Southeast Asia and the Oceanic region show that mutant *pfmdr1* is associated with an increased risk of CQ treatment failure, however this risk is usually substantially higher in the presence of mutant *pfcrt* [17], [28], [36], [64], [65]. Of note, while mutant *pfcrt* is virtually ubiquitous to CQ treatment failures, mutant *pfmdr1* is often absent ([65] and references therein). Functional assays have yet to be developed to test whether *pfmdr1* can directly reduce drug toxicity, or instead is associated with CQR because of its non-random association with mutant *pfcrt*, which potentially could relate to improved parasite fitness [66].

We note that in our study, *pfmdr1* cannot account for differences in the extent to which mutant *pfcrt* affects CQ response, as both the resistant 3D7 and the tolerant D10 mutants (3D7^7G8^ and D10^7G8^ respectively) share the same wild-type *pmfdr1* haplotype (Table 1). The highly resistant GC03 mutants (GC03^7G8^) differ in having the *pfmdr1* N1042D mutation that in allelic exchange studies had no impact on CQ response (although it did affect a number of other antimalarials including QN, mefloquine and ART; [21]). Clear evidence that mutant forms of PfCRT and PfMDR1 can combine in a region-specific manner to create higher levels of drug resistance comes from the recent study by Sa *et al*. [67], showing that the 7G8 South American haplotypes of these two determinants produce high-level resistance to mdADQ. This study also found that the Asian/African Dd2 haplotype of PfCRT was associated with high level CQR with minimal apparent contribution from variant PfMDR1 haplotypes.

Why has no gene other than *pfmdr1* been found associated with CQR? In the case of the HB3×Dd2 genetic cross where mutant *pfcrt* was clearly the primary determinant, evidence that modulatory factors must exist was provided by the 2.7-fold spread in CQ IC_50_ values observed among the CQ-resistant progeny [13]. Such factors may be present within the 36 kb CQR-associated linkage group harboring *pfcrt* [10], [68], or potentially might already be present in the HB3 parent, thereby rendering this competent for CQR and masking the inheritance of a secondary determinant [8]. To test the latter hypothesis, we attempted to introduce mutant *pfcrt* into the HB3 strain, but were unable to obtain integrants in three independent transfection experiments (data not shown). Independent genomic approaches analyzing linkage disequilibrium in CQ-resistant isolates have also failed to identify any gene besides *pfcrt* [14]–[16], [69], as elaborated upon below.

The genetic identity of these secondary determinants associated with CQR may reflect the geographic distribution of distinct PfCRT haplotypes around the globe [19]. Indeed, the PfCRT 7G8 haplotype found in South America and the Pacific is typically associated with PfMDR1 N1042D/D1246Y (±S1034C), whereas the PfCRT Dd2 haplotype common to Asia and Africa is often associated in CQ-resistant isolates with PfMDR1 N86Y [43], [50]. Identifying additional genetic determinants has been complicated by the complexity of performing genome-wide association studies with large numbers of culture-adapted parasite lines from different geographic regions and comparing these to parasite drug responses [50], [70]. Major advances have recently been achieved in a seminal study by Mu *et al.* [69], who performed genome-wide association studies with a 3,000 single nucleotide diversity array probed with DNA from189 culture-adapted *P. falciparum* lines from Africa, Asia, Papua New Guinea and South America, and compared their genetic diversity with CQ response. When accounting for local population structures, the authors found associations between CQ response and changes in *pfcrt, pfmdr1*, and surprisingly a putative tyrosine kinase (PF11_0079). These associations could only readily be discerned in African populations where a sufficient number of CQ-sensitive strains could be identified; as opposed to South American, Asian and Papua New Guinean strains where mutant *pfcrt* remained at a high prevalence. Of the genes listed above, *pfcrt* stood out as being one of handful of genes in the parasite genome that were apparently under very substantial selection pressure in all three populations studied - Asia, Africa and South America. No other genes were convincingly associated with CQR, even though a number of genes potentially involved in drug transport (including the putative drug/metabolite transporter PF14_0260, and the ABC transporters PF13_0271 and PFA0590w) were found to be under lesser selection pressure in local populations. We note that evidence of selection was also observed in genes adjacent to *pfcrt*, although these may simply represent genetic hitchhiking and insufficient time for genetic recombination to have disrupted these associations.

Our conclusion from these studies is that mutant *pfcrt* has been the dominant genetic force that has driven CQR across the globe, with some degree of participation from mutant *pfmdr1*, and that even the phenotype of CQ tolerance observed herein in D10 parasites expressing mutant *pfcrt* would appear sufficient to confer substantial levels of viability during a course of CQ treatment. This level of protection against drug onslaught, while appearing modest *in vitro*, appears to have sufficed for selection and rapid mobility through parasite populations subjected to CQ treatment. Experiments to define secondary determinants that can augment CQR would require, as an example, deeper sequence coverage of the set of 189 genotypically and phenotypically characterized isolates mentioned above [69], followed by quantitative trait loci analysis that computationally subtracted the dominant effect of *pfcrt* to identify potential residual associations in local parasite population structures.

Other hypothesis-driven approaches to identify secondary parasite factors could involve investigations into the function of mutant PfCRT and the cellular basis of CQ mode of action. Recent studies based on heterologous expression of codon-harmonized, surface-expressed PfCRT in *Xenopus laevis* oocytes have recently provided compelling evidence that mutant PfCRT can transport CQ [71], a finding consistent with earlier evidence from *Pichia pastoris* and *Dictyostelium discoideum* [72], [73]. The *Xenopus* study also identified peptides that could interfere with transport of radiolabeled CQ through mutant PfCRT, raising the possibility that PfCRT is involved in transport of certain peptide sequences out of the DV and into the cytoplasm ([74] and references therein). Secondary factors could potentially alter the kinetics of peptide production (resulting from hemoglobin proteolysis in the DV) or their translocation into the parasite cytosol and subsequent conversion into amino acids that can be incorporated into newly synthesized proteins.

Other potential factors could relate to the tri-peptide glutathione (GSH) and redox regulation. Interestingly, an earlier study by Ginsburg and colleagues reported that altering the intracellular levels of GSH caused a corresponding shift in CQ susceptibility in *P. falciparum* [75]. Work from these authors led to the hypothesis that GSH could degrade iron-bound heme (a toxic byproduct of hemoglobin degradation) that might be released into the parasite cytosol as a result of CQ action [76]. Further support for a relationship between GSH and levels of CQR was recently obtained following the genetic disruption of the *P. falciparum* gene PfMRP (PFA0590w), whose ABC transporter product has been localized to the parasite surface. These knockout parasites, generated in the CQ-resistant W2 strain, accumulated more radioactive GSH and CQ and became less resistant to CQ as well as several other antimalarials [77]. Indirect additional evidence of a potential link between CQR and GSH comes from the recent report that PfCRT homologs in *Arabidopsis thaliana* can mediate GSH transport when assayed in *Xenopus* oocytes [78]. Collectively, these data suggest that GSH homeostasis is related to CQR, and possibly to PfCRT, in a strain-dependent manner. A multifactorial, and potentially region-specific basis for these differences would have precluded their identification to date. Further investigations into parasite cell biology, employing genomic, proteomic and metabolomic studies to compare CQ response phenotypes within regional populations, are warranted to identify these molecules and their determinants. French Guinea may well provide an ideal set of geographically restricted isolates in which to define these factors, because of its complex history of antimalarial drug usage and the existence of mutant *pfcrt* strains with both resistance and tolerance phenotypes.

## Materials and Methods

### Ethics statement

Informed consent was not required for this study as the collection of samples from malaria patients for drug susceptibility testing are part of the French national recommendations for the care and surveillance of malaria. As the Pasteur Institute French Guiana laboratory is the regional malaria reference center, blood samples are sent to the laboratory by practitioners (from health centers, private medical offices and hospitals) for drug susceptibility testing, as part of the national regular medical surveillance. This included *in vitro* drug susceptibility testing and assessments of molecular markers. This research is mandated by the French Ministry of Health, and has been approved by the Institutional Review Boards of the Pasteur Institute in Paris and in French Guiana.

### Plasmid constructs

*pfcrt* plasmid inserts were assembled from two contiguous sequences. The first 800 bp sequence, spanning 0.5 kb of the *pfcrt* 5′ UTR (denoted Δ5′) through to the intron 1/exon 2 junction (nucleotides 22960–23747 of the GenBank accession number AF030694), was amplified from Dd2 genomic DNA with the primers p251 and 10AE1-3′A (a list of these and all other primers used in this study is provided in Table S2). A 2.1 kb fragment corresponding to *pfcrt* exons 2–13 and the 3′ UTR of the *P. yoelii* ortholog *pycrt* (termed Py3′) was released following *Avr*II/*Bam*HI digestion of the plasmids pBSD/AE123 -7G8, -GC03, and -SC01 (the latter has the Dd2 sequence) [31]). These two sequences were assembled in pCR2.1 (Invitrogen) to generate a 2.9 kb *pfcrt* fragment containing Δ5′, exon 1, intron 1, exons 2–13, and Py3′. This insert was subcloned as a *Sac*II*/Bam*HI fragment into the pCAM-BSD transfection plasmid. This plasmid expresses the *bsd* selectable marker, which is under control of a 0.6 kb *P. falciparum* calmodulin (*cam*) 5′ UTR and a 0.6 kb *P. falciparum hrp2* 3′ UTR. The resulting 7.2 kb plasmids were designated pBSD-7G8, pBSD-GC03, and pBSD-Dd2.

### Parasite transfections and DNA analysis

The *P. falciparum* 3D7, D10, and GC03 strains were cultured in human erythrocytes, transfected as described [21], and selected with 2.0 mg/ml blasticidin HCl (Invitrogen). Upon integration, recombinant parasites were cloned by limiting dilution and identified using Malstat assays [31]. The isolates from French Guyana were collected from malaria patients referred to the reference malaria laboratory of the Pasteur Institute of Guyana, in Cayenne, France. Each year this work was reviewed and approved by the Pasteur Institute Surveillance Committees of Guyana and Paris. The institutional review board of the Columbia University Medical Center also reviewed and approved the *P. falciparum* culture work.

PCR-based detection of plasmid integration into transfected parasites (Figure 1) used the *pfcrt* 5′ UTR-specific primer p1, the *pfcrt* exon 5-specific primer p2, the Py3′-specifc primer p3, the *pfcrt* intron 2-specific primer p4, and the plasmid-specific primer p5. For Southern blot analysis, 1 µg of DNA was digested with *Eco*RI*/Bgl*II, electrophoresed, and transferred onto nylon membranes. Hybridizations were performed with a hexamer-primed [^32^P]-labeled probe prepared from the 0.8 kb fragment spanning Δ5′, exon1 and intron 1, and released following *Sac*II/*Avr*II digestion of the transfection plasmid pBSD-Dd2. The full-length sequence of *pfcrt* was determined from the complete coding sequence amplified from cDNA using the primers p251+BB116C and sequenced internally with the primers CF5C, BB84, AF12, AB22, AB25, and BB116B. For sequencing of the upstream *pfmdr1* polymorphic residues at positions 86 and 184, genomic DNA was amplified with the primers p423+p231, and the resulting 0.7 kb products were sequenced with p231. For the downstream polymorphic residues at positions 1034, 1042, and 1246, the 0.8 kb amplification product of p426+p215 was sequenced with p238. *pfmdr1* copy number was measured by Taqman quantitative real-time PCR and quantified with the ΔΔCt method as described elsewhere [79]. Genomic DNA samples were run twice in triplicate.

### Quantitative real-time RT-PCR assays

The expression of *pfcrt* in the recombinant clones was assessed by quantitative real-time PCR assays performed with the QuantiTect SYBR Green PCR Kit (Qiagen) on an Opticon2 (BioRad). Expression from the different alleles (endogenous and genetically introduced) was analyzed utilizing primers specific for the two different 3′ UTRs, designated Py3′ and Pf3′. For the loci containing Py3′, the primers p1752 and p1753 were used to generate a 182 bp amplicon. For the locus containing Pf3′, a 191 bp amplicon was generated using the primers p1754 and p1756. PCR conditions were optimized so that the relative efficiencies of the Pf3′ and Py3′ amplifications were equal. Reactions were performed in 25 mL volumes with 300 nM of each primer, 3 mM Mg^2+^, and 1/80^th^ of the oligo(dT) primed cDNA generated from 1.5 µg of total RNA. As a control for each sample, a 150 bp amplicon of β-actin was amplified using the primers A129 and A130, using the same conditions as for Py3′ and Pf3′ except that the Mg^2+^ concentration was 3.5 mM. All amplifications were performed with 15 minutes of hot start at 95°C, followed by 40 cycles of denaturing for 30 seconds at 95°C, annealing for 30 seconds at 49°C, and extension for 30 seconds at 62°C. Melting curve analysis was performed for each assay to verify that a single melting peak was produced, indicating a single specific PCR product for each reaction. A standard curve for each reaction was generated with 10-fold serial dilutions of genomic DNA, spanning the range of 5 to 5×10^5^ genome copies). This genomic DNA was prepared from D10^C^, a recombinant clone shown by Southern hybridization to have a single copy of each locus (Py3′ and Pf3′, Figure 1B). Each sample was run in triplicate on three separate occasions.

### Protein analysis

Protein extracts were prepared from sorbitol-synchronized trophozoite-stage parasites. For each sample, protein from ∼1×10^6^ parasites was loaded per well, electrophoresed on 12% SDS-PAGE gels, and transferred onto polyvinylidene difluoride membranes. Membranes were probed with rabbit anti-PfCRT antibodies (diluted 1∶2,500) [11], followed by incubation with horseradish peroxidase-conjugated donkey anti-rabbit IgG (1∶10,000; Amersham Biosciences). Rabbit anti-PfERD2 antibodies (diluted 1∶1,000) [80] were used as an independent loading control. Bands were visualized by enhanced chemiluminescence (Amersham Biosciences) and quantified by densitometric analysis of autoradiograph data using NIH ImageJ 1.38× (http://rsb.info.nih.gov/ij). PfCRT band intensities were normalized against the PfERD2 bands to correct for minor differences in protein loading.

### *In vitro* antimalarial drug assays

Parasite susceptibilities to antimalarial drugs were measured *in vitro* by [^3^H]-hypoxanthine incorporation assays, as described [81]. Briefly, predominately ring-stage cultures were seeded in duplicate in 96-well plates at 0.4% parasitemia and 1.6% hematocrit. Parasites were exposed to a range of drug concentrations, or no drug controls, for 72 hr, with 0.5 µCi per well of [^3^H]-hypoxanthine added at the 48 hr time point. IC_50_ and IC_90_ values were extrapolated by linear regression, as described [81]. Compounds were tested in duplicate on 4–11 separate occasions for CQ and mdCQ and 3–12 separate occasions for the other drugs. In some assays, VP was included at 0.8 µM final concentration. Statistical analyses comparing mutant *pfcrt*-modified lines against recombinant control lines of the same genetic backgrounds were performed using one-way ANOVA with a Bonferroni post-hoc test for CQ and mdCQ, or unpaired student *t* tests for quinine (QN), artemisinin (ART), monodesethyl-amodiaquine (mdADQ), lumefantrine (LMF), and piperaquine (PIP).

### *In vitro* recrudescence assays

Parasites were assayed for their ability to grow under short-term exposure to high CQ concentrations. Predominately ring-stage cultures were seeded in 96-well plates at 0.2% parasitemia and 1.6% hematocrit. Parasites were exposed for 6 days to no drug, 50 nM CQ, or 80 nM CQ, with daily media changes. Drug pressure was then removed on day 7 and parasite growth was measured using Malstat assays ([31]). From days 7 through 30, media changes and Malstat assays were performed every two days, and the cultures cut 1∶2 into fresh erythrocytes weekly until the detection of positive wells. As part of this experiment, cultures of 3D7^7G8-1^ and D10^7G8-1^ were exposed to 50 nM CQ for 6 days and maintained until parasites became microscopically detectable, at days 15 and 20 respectively. These CQ-pretreated cultures were assayed for recrudescence alongside 7G8, 3D7^C^, 3D7^7G8-1^, D10^C^, D10^7G8-1^, GC03^C^, and GC03^7G8-1^. Data were pooled from two independent experiments in which each line was assayed in duplicate for the no drug controls and in triplicate for the 50 nM and 80 nM CQ treatments.

### Malstat assays

These were performed as described [82], with minor modifications. Briefly, 100 µL of Malstat reagent was added to 50 µL of culture supernatant and incubated for 1 hr. Absorbance at 595 nM was measured on a VICTOR^3^ Multilabel Plate Reader (Perkin-Elmer). Wells positive for parasite growth were identified based on absorbance values greater than twice those obtained from control wells with uninfected erythrocytes. Positive wells were verified by microscopic evaluation of Giemsa-stained thin smears.

### Gene identification numbers

*pfcrt*: MAL7P1.27; *pfmdr1*: PFE1150w; *pycrt*: PY05061; b-actin: PFL2215w. *Pfmrp*: PFA0590w. All numbers are from www.plasmodb.org.

## Supporting Information

Figure S1 Measurements of the degree of verapamil reversibility of chloroquine and monodesethyl- chloroquine in *pfcrt*-modified and control *Plasmodium falciparum* lines.
(0.17 MB PDF)

Figure S2 Ratios of chloroquine IC_90_ to IC_50_ values in *pfcrt*-modified lines.
(0.60 MB PDF)

Table S1 Antimalarial IC_50_ and IC_90_ values of *pfcrt*-modified and reference lines.
(0.07 MB PDF)

Table S2 List of oligonucleotide primers used in this study.
(0.05 MB PDF)
