# Supplementary material for: Identification of a Mutant PfCRT-Mediated Chloroquine Tolerance Phenotype in Plasmodium falciparum
Source: PLoS Pathog. 2010 May 13;6(5):e1000887. doi: 10.1371/journal.ppat.1000887 (PMC2869323; doi:10.1371/journal.ppat.1000887)
Supplement: Figure S1 — Measurements of the degree of verapamil reversibility of chloroquine and monodesethyl- chloroquine in pfcrt-modified and control Plasmodium falciparum lines. (0.17 MB PDF) [file ppat.1000887.s001.pdf]

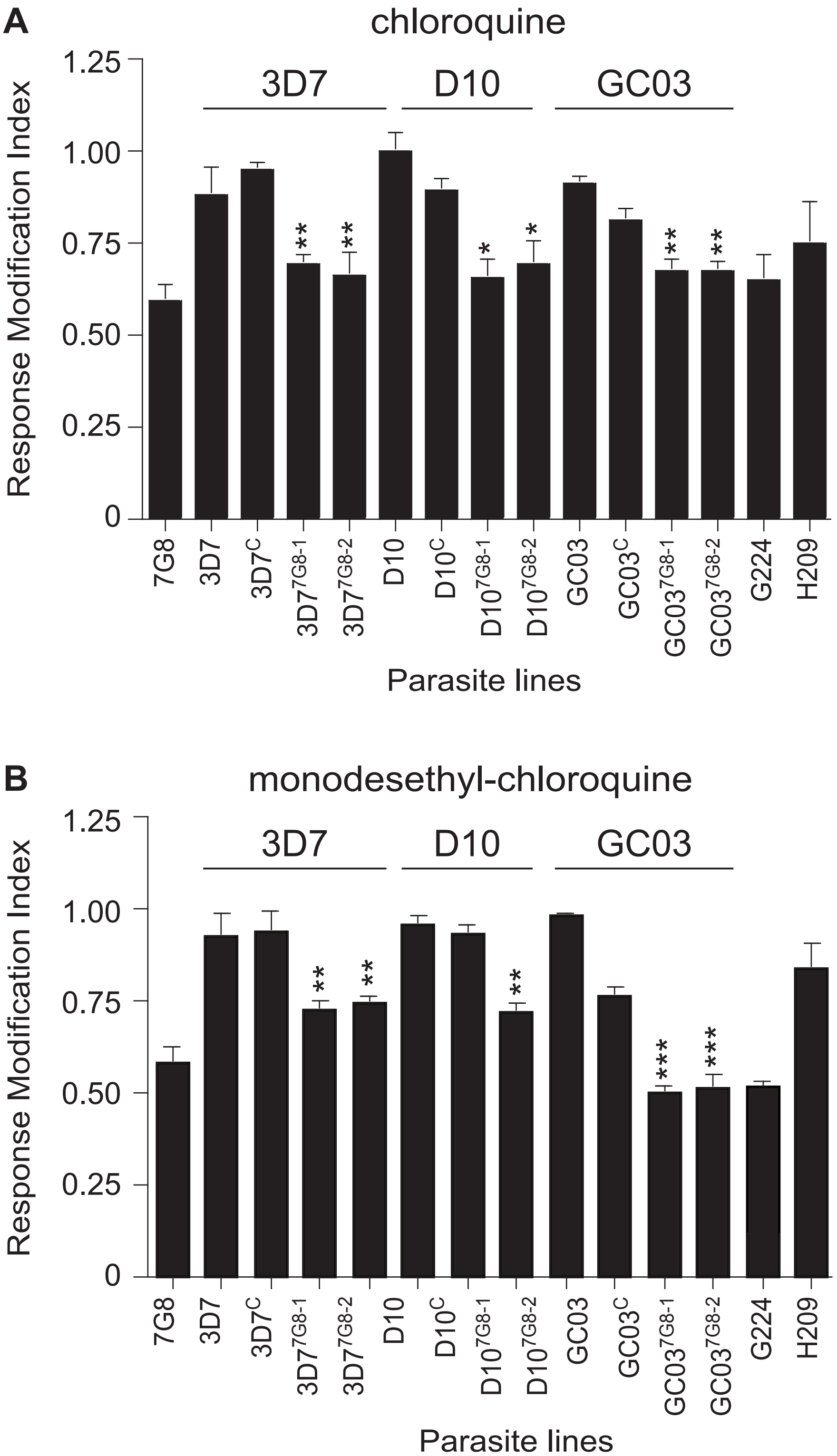


**Figure S1. Measurements of the degree of verapamil reversibility of chloroquine and monodesethyl-chloroquine in *pfcrt*-modified and control *Plasmodium falciparum* lines.** The Response Modification Index (RMI), calculated as the ratio of the $IC_{50}$ in the presence of verapamil (VP) to that in the absence of VP, quantifies the degree of VP reversibility. RMI mean±SEM values were obtained from an average of 6 (range 3–11) independent assays performed in duplicate. Unpaired students *t* tests were used to compare the RMI of the *pfcrt*-modified lines to their respective recombinant controls. *$P$<0.05; **$P$<0.01; ***$P$<0.001. In the 3D7, D10, and GC03 genetic backgrounds, mutant *pfcrt* conferred VP reversibility of the (A) chloroquine and (B) monodesethyl-chloroquine responses.
