# Supplementary material for: Identification of a Mutant PfCRT-Mediated Chloroquine Tolerance Phenotype in Plasmodium falciparum
Source: PLoS Pathog. 2010 May 13;6(5):e1000887. doi: 10.1371/journal.ppat.1000887 (PMC2869323; doi:10.1371/journal.ppat.1000887)
Supplement: Figure S2 — Ratios of chloroquine IC90 to IC50 values in pfcrt-modified lines. (0.60 MB PDF) [file ppat.1000887.s002.pdf]

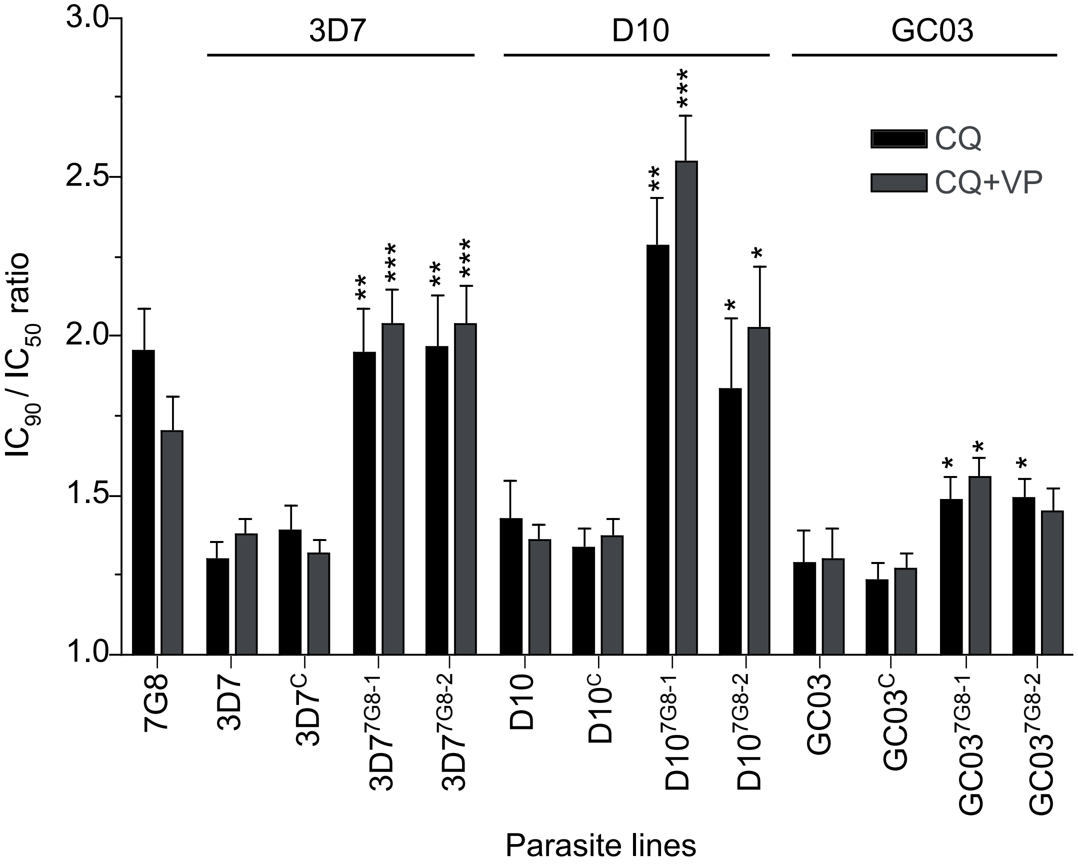


**Figure S2. Ratios of chloroquine $IC_{90}$ to $IC_{50}$ values in *pfcrt*-modified lines.** Ratios are presented as means±SEM, which were calculated from an average of 7 independent assays (range 4–10) performed in duplicate. Analysis of the chloroquine (CQ) $IC_{90}/IC_{50}$ ratios for the different lines revealed a very different set of responses between the three CQ-sensitive genetic backgrounds. In 3D7, both mutant *pfcrt* clones revealed mean ratios of 2.0, the same as 7G8 and noticeably greater than the ratios of 1.3–1.4 observed with $3D7^{C}$ (*P*<0.01) and 3D7. For D10, where mean CQ $IC_{50}$ values were unchanged compared to $D10^{C}$ (Table S1), the ratios were 1.8–2.3, a significant increase over the ratios of 1.3–1.4 again observed with $D10^{C}$ (*P*<0.01) and D10. Thus, in both these backgrounds the relatively modest increase in CQ $IC_{50}$ values appeared to be compensated by a substantial increase in the ability of these parasites to withstand high CQ concentrations. For the GC03 mutants, where the baseline CQ $IC_{50}$ values were already high, the increase in the $IC_{90}$ / $IC_{50}$ ratio was more modest (1.5 for both $GC03^{7G8\text{-}1}$ and $GC03^{7G8\text{-}2}$ vs. 1.2–1.3 for the control CQ-sensitive lines $GC03^{C}$ (*P*<0.05) and GC03), suggesting that the expression of mutant *pfcrt* rendered GC03 parasites intrinsically more resistant to CQ than was the case for 3D7 or D10 parasites. Statistical comparisons comparing mutant *pfcrt*-modified lines against recombinant control lines of the same genetic backgrounds were performed using one-way ANOVA with a Bonferroni post-hoc test.
