# Supplementary material for: Identification of a Mutant PfCRT-Mediated Chloroquine Tolerance Phenotype in Plasmodium falciparum
Source: PLoS Pathog. 2010 May 13;6(5):e1000887. doi: 10.1371/journal.ppat.1000887 (PMC2869323; doi:10.1371/journal.ppat.1000887)
Supplement: Table S1 — Antimalarial IC50 and IC90 values of pfcrt-modified and reference lines. (0.07 MB PDF) [file ppat.1000887.s003.pdf]

Table S1. Antimalarial IC_{50} and IC_{90} values of *pfcrt*-modified and reference lines*

	Parasite Lines (lab clone name listed below)																
Drug	7G8	3D7	3D7^{C} BXB2	3D7^{7G8-1} CZ1	3D7^{7G8-2} CZ4	D10	D10^{C} BWG10	D10^{7G8-1} BY4	D10^{7G8-2} BY6	GC03	GC03^{C} DW1	GC03^{7G8-1} DX1	GC03^{7G8-2} DX3	C6^{7G8}	Dd2	G224**	H209**
CQ IC_{50}	189.6±13.5	30.4±2.6	29.1±2.3	83.7±13.9	79.4±10.8	40.7±2.3	45.1±3.1	62.9±10.8	70.7±16.0	31.3±0.6	27.4±2.8	130.8±7.0	128.5±8.2	130.1±7.3	277.6±38.3	52.4±7.9	34.8±6.8
p value				P<0.001	P<0.001			ns	ns			P<0.001	P<0.001	P<0.001			
# of assays	10	7	10	6	6	5	6	6	6	4	5	9	9	4	7	6	6
CQ+VP IC_{50}	101.7±7.0	28.7±2.4	23.1±3.3	61.3±7.0	56.8±5.8	42.5±3.5	44.8±3.1	41.7±5.5	49.9±8.5	28.7±2.3	24.1±0.9	78.6±5.3	83.8±7.5	84.7±6.3	76.2±12.6	33.2±4.7	22.5±2.8
p value				P<0.001	P<0.001			ns	ns			P<0.001	P<0.001	P<0.001			
% reversal	46.4%	5.7%	20.4%	26.8%	28.5%	-4.3%	0.7%	33.7%	29.5%	8.4%	11.8%	39.9%	34.8%	34.9%	72.6%	36.6%	35.4%
# of assays	10	7	10	6	6	5	6	6	6	4	5	9	9	4	7	6	6
CQ IC_{90}	363.1±27.28	39.2±3.0	39.6±2.5	157.7±21.8	154.5±23.8	57.8±4.7	60.5±4.7	148.2±35.4	125.8±27.9	40.4±2.7	33.5±3.0	206.4±12.3	204.1±15.6	216.2±6.9	387.5±38.0	122.6±26.8	44.3±7.0
p value				P<0.001	P<0.001			ns	ns			P<0.001	P<0.001	P<0.001			
# of assays	10	7	10	6	6	5	6	6	6	4	5	9	9	4	7	6	6
CQ+VP IC_{90}	171.6±14.2	34.9±2.5	32.6±3.5	123.0±12.5	113.5±8.4	57.3±3.9	61.0±4.7	104.1±11.5	100.7±18.5	37.4±3.1	30.4±0.2	117.4±8.5	123.0±9.3	120.9±10.1	97.2±13.7	58.3±5.6	36.5±2.6
p value				P<0.001	P<0.001			ns	ns			P<0.001	P<0.001	P<0.001			
% reversal	52.7%	11.0%	17.8%	22.0%	26.5%	1.0%	-0.8%	29.8%	20.0%	7.5%	9.3%	43.1%	39.7%	44.1%	74.9%	52.4%	17.5%
# of assays	10	7	10	6	6	5	6	6	6	4	5	9	9	4	7	6	6
mdCQ IC_{50}	855.6±71.7	42.4±3.4	42.8±2.8	437.3±22.1	456.3±27.7	75.9±8.5	70.3±14.9	334.8±39.2	nd	35.3±1.4	49.6±10.0	467.6±75.8	464.9±83.0	nd	1287±131.0	348.9±45.8	69.5±8.8
p value				P<0.001	P<0.001			P<0.01				P<0.01	P<0.01				
# of assays	11	5	10	7	7	4	4	7		4	5	5	5		10	8	8
mdCQ+VP IC_{50}	494.7±44.5	44.4±7.2	36.0±2.4	326.7±14.1	354.1±18.6	69.3±7.0	63.2±12.7	235.2±22.0	nd	34.6±14.9	38.6±8.7	233.9±40.1	244.4±57.5	nd	186.5±54.7	179.5±16.9	39.5±1.4
p value				P<0.001	P<0.001			P<0.01				P<0.05	P<0.05				
% reversal	42.2%	-4.7%	15.8%	25.3%	22.4%	8.7%	10.2%	29.7%		2.0%	22.3%	50.0%	47.4%		85.5%	51.5%	43.2%
# of assays	11	5	10	7	7	4	4	7		4	5	5	5		5	3	3
mdCQ IC_{90}	1639±184.1	73.7±4.0	78.6±6.0	798.7±28.9	826.0±30.5	99.3±9.9	98.0±11.8	670.4±50.0	nd	52.1±2.4	78.8±7.5	683.6±109.0	723.4±117.7	nd	1909±136.3	779.1±59.5	116±10.2
p value				P<0.001	P<0.001			P<0.01				P<0.01	P<0.01				
# of assays	9	5	10	7	7	4	4	7		4	5	5	5		10	8	8
mdCQ+VP IC_{90}	952.9±121.4	71.90±7.5	66.4±4.6	641.6±32.4	665.9±37.4	100.3±10.6	104.6±19.7	512.2±27.1	nd	51.1±3.8	60.2±6.4	371.9±64.2	371.2±71.8	nd	266.8±60.2	119.0±15.5	72.6±3.8
p value				P<0.001	P<0.001			P<0.01				P<0.01	P<0.01				
% reversal	41.9%	2.4%	15.5%	19.7%	19.4%	-1.0%	-6.7%	23.6%		1.9%	23.7%	45.6%	48.7%		86.0%	51.5%	37.7%
# of assays	11	5	10	7	7	4	4	7		4	5	5	5		5	3	3
QN IC_{50}	160.9±31.6	52.4±6.7	57.1±4.3	51.6±13.8	44.5±10.3	56.8±9.3	71.8±12.8	77.5±5.9	nd	nd	118.3±17.9	77.3±14.3	78.9±17.7	nd	275.2±78.1	146.9±1.5	405.0±40.2
p value				ns	ns			ns				ns	ns				
# of assays	8	5	10	4	4	5	4	3			5	5	5		5	3	3
ART IC_{50}	18.6±1.3	38.8±5.5	43.5±5.2	29.3±4.1	nd	39.0±6.8	47.4±9.6	23.2±5.3	nd	nd	29.3±3.4	15.0±1.2	15.9±4.2	nd	39.0±9.9	24.8±1.4	56.2±2.0
p value				ns				P<0.05				P<0.01	P<0.01				
# of assays	10	7	10	7		7	7	7			3	3	3		3	3	3
mdADQ IC_{50}	67.4±4.7	19.9±3.6	19.7±3.2	32.7±3.8	nd	26.3±3.1	28.9±3.6	30.9±4.3	nd	nd	19.7±0.7	52.2±8.3	56.9±12.5	nd	51.6±13.2	54.0±2.2	49.0±1.8
p value				P<0.05				ns				P<0.01	P<0.01				
# of assays	12	7	12	7		7	7	7			5	5	5		5	3	3
LMF IC_{50}	26.5±4.4	73.2±6.1	69.4±2.5	53.6±6.8	nd	66.6±5.2	68.5±4.8	38.8±6.1	nd	nd	46.2±3.4	29.8±2.8	28.3±2.1	nd	35.2±5.1	8.9±0.3	15.5±0.4
p value				P<0.05				P<0.01				P<0.01	P<0.01				
# of assays	11	6	11	6		6	6	6			5	5	5		5	3	3
PIP IC_{50}	20.2±1.1	15.6±1.1	18.0±1.6	17.2±1.6	nd	18.3±2.2	25.5±4.2	14.6±2.5	nd	nd	19.5±2.4	22.3±3.3	21.1±3.6	nd	31.1±6.9	7.3±0.8	11.6±0.7
p value				ns				P<0.05				ns	ns				
# of assays	12	7	12	7		7	7	7			5	5	5		5	3	3

*IC_{50} and IC_{90} values were derived by linear extrapolation of drug inhibition data generated from 72 h [3H]-hypoxanthine incorporation assays. All lines were tested at least three times in duplicate against each antimalarial drug Values indicate mean ± SEM, shown in nM. Statistical comparisions of the mutant clones against the corresponding recombinant control were made by one-way ANOVA with Bonferroni posttests (CQ and mdCQ) and unpaired students t test for QN, ART, mdADQ, LMF, and PIP. CQ, chloroquine; VP, verapamil (used at 0.8 μM); mdCQ, monodesethylchloroquine; QN, quinine; ART, artemisinin; mdADQ, monodesethylamodiaquine; LMF, lumefantrine; PIP, piperaquine.

**G224 and H209 were also assayed against the artemisinin derivatives artemether, artesunate and dihydroartemisinin (4 independent assays tested in duplicate). Mean±SEM IC_{50} values were - artemether: 2.1±0.8 nM for G224 and 2.4±0.4 nM for H209; artesunate: 7.0±2.6 nM for G224 and 9.7±3.2 nM for H209; and dihydroartemisinin: 2.2±0.5 nM for G224 and 2.5±0.4 nM for H209.
