# Supplementary material for: Identification of a Mutant PfCRT-Mediated Chloroquine Tolerance Phenotype in Plasmodium falciparum
Source: PLoS Pathog. 2010 May 13;6(5):e1000887. doi: 10.1371/journal.ppat.1000887 (PMC2869323; doi:10.1371/journal.ppat.1000887)
Supplement: Table S2 — List of oligonucleotide primers used in this study. (0.05 MB PDF) [file ppat.1000887.s004.pdf]

Table S2. List of oligonucleotide primers used in this study

Primer	Gene	Location	Sequence
p251	*pfcrt*	5' UTR, fwd	5'-caaccgcggaaggtaccCCCCAAGTTGTACTGCTTCTAAG*
10AE1-3'A	*pfcrt*	intron 1-exon 2, rev	5'-GGCGTGAGCCATCTGTTAAGGTC
p1 (10 5' F1)	*pfcrt*	5' UTR, fwd	5'-ACATATATTTTTAATAAATGTCCTCC
p2 (AB17)	*pfcrt*	exon 5, rev	5'-TACAACAATAATAACTGCTCCGAG
p3 (p173)	*pycrt*	3' UTR, rev	5'-GTATCAAACATAGAAATACACCGT
p4 (T3)	pBluescript	multiple cloning site	5'-CAATTAACCCTCACTAAAGGG
p5 (10 5' R5)	*pfcrt*	intron 2, rev	5'-GTAATGTTTTATATTGGTAGGTGG
p6 (CF5C)	*pfcrt*	exon 1	5'-AATTCAAGCAAAAATGACGAGCG
CF5B	*pfcrt*	5' UTR, fwd	5'-CCGTTAATAATAAATACACGCAG
AB25	*pfcrt*	exon 13, rev	5'-CGACGTTGGTTAATTCTCCTTCGG
BB84	*pfcrt*	exon 3, rev	5'-GACTGAACAGGCATCTAACATGG
AF12	*pfcrt*	exon 2, fwd	5'-AGATGGCTCACGTTTAGGTGGAGG
AF22	*pcfrt*	exon 8, fwd	5'-GTGATGATTGTGACGGAGCATGG
BB116C	*pfcrt*	3' UTR, rev	5'-CTATTATCATGATTGAAGAATGG
BB116B	*pfcrt*	3' UTR, rev	5'-TTATAAAGTGTAATGCGATAGC
p423	*pfmdr1*	fwd	5'-AAAGATGGTAACCTCAGTATCAAAGAAGAG
P231	*pfmdr1*	rev	5'-AGATATTACCTGTACCATAACAATAATA
p426	*pfmdr1*	fwd	5'-ATGATCACATTATATTAAAAAATGATATGACAA
p215	*pfmdr1*	rev	5'-TACGTATTTTTTATATATTCCATCTTGTGC
p238	*pfmdr1*	fwd	5'-TTTCAAACCAATCTGGATCTGCA
p1752	*pfcrt*	exon 11-12, fwd	5'-ATTTCGTAACTTTGTTTGGC
p1753	*pycrt*	3' UTR rev	5'-TAGTTTGTATCTTTATGTGGCA
p1754	*pfcrt*	exon 13, fwd	5'-ATGAAGAAAATGAAGATTCCG
p1755	*pfcrt*	3' UTR, rev	5'-TTCCTTATAAAGTGTAATGCGA
A129	*Pf* β-actin	fwd	5'-AGCAGCAGGAATCCACACA
A130	*Pf* β-actin	rev	5'-TGATGGTGCAAGGGTTGTAA

**Sac*II and *Bst*EII sites are underlined. UTR, untranslated region; fwd, forward; rev, reverse.
